# Effect of the BH3 Mimetic Polyphenol (–)-Gossypol (AT-101) on the *in vitro* and *in vivo* Growth of Malignant Mesothelioma

**DOI:** 10.3389/fphar.2018.01269

**Published:** 2018-11-06

**Authors:** Monica Benvenuto, Rosanna Mattera, Joshua Ismaele Sticca, Piero Rossi, Chiara Cipriani, Maria Gabriella Giganti, Antonio Volpi, Andrea Modesti, Laura Masuelli, Roberto Bei

**Affiliations:** ^1^Department of Clinical Sciences and Translational Medicine, University of Rome Tor Vergata, Rome, Italy; ^2^Department of Experimental Medicine and Surgery, University of Rome Tor Vergata, Rome, Italy; ^3^Department of Experimental Medicine, Sapienza University of Rome, Rome, Italy

**Keywords:** polyphenol, AT-101, BH3 mimetic, malignant mesothelioma, apoptosis, autophagy

## Abstract

Malignant mesothelioma (MM) is a primary tumor arising from mesothelial cells. The survival of MM patients following traditional chemotherapy is poor, thus innovative treatments for MM are needed. (-)-gossypol (AT-101) is a BH3 mimetic compound which possesses anti-tumoral activity by targeting multiple signaling transduction pathways. Several clinical trials employing AT-101 have been performed and some of them are still ongoing. Accordingly, we investigated the *in vitro* effects of AT-101 on cell proliferation, cell cycle regulation, pro-survival signaling pathways, apoptosis and autophagy of human (MM-B1, H-Meso-1, and MM-F1) and mouse (#40a) MM cell lines. In addition, we explored the *in vivo* anti-tumor activities of AT-101 in a mouse model, in which the transplantation of MM cells induces ascites in the peritoneal space. AT-101 inhibited *in vitro* MM cells survival in a dose- and time-dependent manner and triggered autophagy, but the process was then blocked and was coincident with apoptosis activation. To confirm the effect of AT-101 in inducing the apoptosis of MM cells, MM cells were simultaneously treated with AT-101 and with the caspase inhibitor, Z-VAD-FMK. Z-VAD-FMK was able to significantly reduce the number of cells in the subG1 phase compared to the treatment with AT-101 alone. This result corroborates the induction of cell death by apoptosis following treatment with AT-101. Indeed, Western blotting results showed that AT-101 increases Bax/Bcl-2 ratio, modulates p53 expression, activates caspase 9 and the cleavage of PARP-1. In addition, the treatment with AT-101 was able to: (a) decrease the ErbB2 protein expression; (b) increase the EGFR protein expression; (c) affect the phosphorylation of ERK1/2, p38 and AKT; (d) stimulate JNK1/2 and c-jun phosphorylation. Our *in vivo* results showed that the intraperitoneal administration of AT-101 increased the median survival of *C57BL/6* mice intraperitoneally transplanted with #40a cells and reduced the risk of developing tumors. Our findings may have important implications for the design of MM therapies by employing AT-101 as an anticancer agent in combination with standard therapies.

## Introduction

Malignant mesothelioma (MM) is a primary tumor arising from mesothelial cells in the lining of body, in particular, the chest or abdominal area (Carbone et al., 2012).

The development of MM is associated with multi-factorial etiology and consists of a multi-step process, in which genetically modified mesothelial cells are prone to grow, develop a more aggressive phenotype and eventually acquire the ability to metastasize and invade other tissues (Carbone et al., 2012; Benvenuto et al., 2016b).

Malignant mesothelioma shows three histological subtypes: epithelioid, sarcomatoid and biphasic. Epithelioid mesothelioma is characterized by epithelioid cells with large, eosinophilic cytoplasm organized in glandular or pseudoacinar structures. This subtype displays a better prognosis compared to other malignant mesothelioma types. Sarcomatoid mesothelioma is composed of atypical spindle cells. Biphasic (mixed) type presents both epithelioid and sarcomatoid components (Del Gobbo et al., 2014).

Malignant mesothelioma has a long latent period, about 30–40 years but presents a median survival of 7–12 months after the diagnosis (Lemen, 2016). Surgery, chemotherapy and radiotherapy have increased the patient’s quality of life, but the survival of patients with MM remains poor (Favoni and Florio, 2011). The resistance of MM to therapeutics is probably due to the immunosubversion that MM cells induce on immune cells (Izzi et al., 2009). The resistance of MM to conventional therapies and the poor patient survival following traditional chemotherapy have supported the identification of novel molecular targeted therapies for MM treatment (Thellung et al., 2016). Polyphenols can be used as anti-cancer drugs in association with other therapies. Polyphenols have demonstrated to inhibit several signaling pathways in cancer cells, and to induce their death (Benvenuto et al., 2016b).

Gossypol (C_30_H_30_O_8_), chemically known as 1,1′,6,6′,7,7′-hexahydroxy-5,5′-diisopropyl-3,3′-dimethyl (2,2′-binaphthalene)-8,8′-dicarboxaldehyde, is a natural polyphenolic aldehyde produced by a pigment of cotton (*Gossypium* spp.) found in the seeds of plants and in cotton plant by-products, such as cottonseed oil and cottonseed meal flour. (Huang et al., 2006; Câmara et al., 2015). The naturally occurring gossypol is a racemic mixture of two enantiomers, (+)-gossypol and (-)-gossypol (also called AT-101) that exists with different ratios in *Gossypium* species (Tian et al., 2016). Gossypol showed contraceptive, anti-virus, anti-microbial, anti-parasitic, anti-oxidant and anti-tumoral properties. The enantiomer (-)-gossypol has a more potent cytotoxic effect in cancer cells than the (+)-gossypol or racemic gossypol (Keshmiri-Neghab and Goliaei, 2014).

Gossypol is a BH3 mimetic compound (Opydo-Chanek et al., 2017). The Bcl-2 family proteins (Bcl-2, Bcl-xL, Bcl-W, Mcl-1, A1/BFL-1) interact with BH3 proteins, such as Bax or Beclin-1, and regulate various intracellular pathways, including apoptosis and autophagy (Maiuri et al., 2007; Sinha and Levine, 2008; Vela et al., 2013; Benvenuto et al., 2017). Initially, it has been demonstrated that gossypol directly bound Bcl-xL (Kitada et al., 2003). Other studies showed that gossypol was a pan-Bcl-2 inhibitor, capable to inhibit Bcl-2, Bcl-xL, Mcl-1, and Bcl-w (Opydo-Chanek et al., 2017). Gossypol binds to the BH3 binding groove of anti-apoptotic Bcl-2 proteins, thus inhibiting the anti-apoptotic function of Bcl-2, Bcl-xl, and Mcl-1, and inducing apoptosis of cancer cells (Kang and Reynolds, 2009). In addition, gossypol prevents the interaction between Bcl-2 and Beclin-1 at the endoplasmic reticulum, decreases the levels of Bcl-2 and increases Beclin-1 expression by inducing Beclin-1 Atg5-a dependent autophagic pathway in cancer cells (Lian et al., 2011).

In the last years many studies reported the anti-tumoral effects of gossypol in several types of cancer, including leukemia, lymphoma, colon carcinoma, breast cancer, myoma, prostate cancer and others (Gadelha et al., 2014; Keshmiri-Neghab and Goliaei, 2014).

In addition, several clinical trials employing AT-101 have been developed and some trials are still ongoing (Opydo-Chanek et al., 2017; ClinicalTrials.gov, 2018). The phase I/II clinical trials with AT-101 combined with chemotherapy in small cell lung cancer (SCLC), NSCLC, and CLL displayed positive responses (Opydo-Chanek et al., 2017).

In this study, we investigated the anti-tumoral effects of AT-101 in MM. We analyzed the *in vitro* effects of AT-101 on cell proliferation, cell cycle regulation, apoptosis, autophagy and pro-survival signaling pathways in human and mice MM cell lines. Furthermore, we explored the *in vivo* effects of AT-101 in a mouse model (C57BL/6 mice), in which the transplantation of MM cells induces ascites in the peritoneal space. Our findings may have important implications for the design of MM therapies by employing AT-101 as an anticancer agent in combination with standard therapies.

## Materials and Methods

### Reagents

DMSO, Sulforhodamine B (SRB), Hoechst 33342 and Pristane (2,6,10,14-Tetramethylpentadecane) were purchased from Sigma-Aldrich (Milan, Italy). (-)-gossypol (AT-101) was provided from Selleck Chemical (Munich, Germany). Z-VAD-FMK was purchased from Calbiochem (San Diego, CA, United States). Antibodies against AKT, phospho-AKT, Bax, Bcl-2, JNK/SAPK1, JNK/SAPK (pT183/pY185), p38a/SAPK2a, and p38 MAPK (pT180/pY182) were obtained from BD Pharmingen (BD Biosciences, San Jose, CA, United States). Antibodies against caspase 9, caspase 8, activated caspase 3, c-Jun, phospho-c-Jun, were obtained from Cell Signaling Technology (MA, United States). ERK1/2 (C-14), phospho-ERK (E-4), p53 (DO-1) and PARP-1 (F-2) were obtained from Santa Cruz Biotechnology (CA, United States). Anti-ErbB2 and anti-EGFR antisera were provided by Dr. M. H. Kraus (University of Alabama, Birmingham, AL, United States). Antibodies against Beclin-1 and p62/SQSTM1 were obtained from Abcam (Cambridge, United Kingdom) and the anti-LC3 antibody was purchased from Novus Biologicals (Littleton, CO, United States). The goat anti-rabbit IgG Alexa fluor-594-conjugated secondary antibody was from Invitrogen (Milan, Italy). Rabbit polyclonal antibody against actin and tubulin and the goat anti-mouse or -rabbit IgG peroxidase conjugated secondary antibodies were from Sigma-Aldrich.

### Cell Lines and Treatments

Human (MM-B1, H-Meso-1, MM-F1) and mouse (40 and #40a) MM cell lines were maintained in DMEM (Dulbecco’s modified Eagle’s medium) containing 10% fetal bovine serum, 100 U/ml penicillin and 100 μg/ml streptomycin (complete medium). Cells were grown at 37°C in a humidified incubator with an atmosphere of 5% CO_2_. The #40a cell line derives from the 40 cell line after two passages in the peritoneal cavity of C57BL/6 mice. These passages allow the selection of cells which reproducibly form ascites when intraperitoneally injected in the mice. H-Meso-1 cells have an epithelial morphology, while MM-B1 and MM-F1 cells have biphasic and sarcomatous features, respectively (Palumbo et al., 2008). The 40 cell line which has an epithelial morphology (Goodglick et al., 1997) was kindly provided by Dr. Agnes Kane (Department of Pathology and Laboratory Medicine, Brown University, Providence, Rhode Island).

AT-101 was dissolved in DMSO. For treatments, cells were incubated for the indicated times in the presence of AT-101 (dose range: 1.56–25 μM) or vehicle control (DMSO ≤ 0.1%).

### Sulforhodamine B (SRB) Assay

Cells were seeded at 5 × 10^3^/well in 96-well plates and incubated at 37°C to allow cell attachment. After 24 h, the medium was changed and the cells were treated with AT-101 (1.56–3.13–6.25–12.5–25 μM) or DMSO and incubated for 24, 48, and 72 h. Cells were then fixed with cold trichloroacetic acid (final concentration 10%) for 1 h at 4°C. After four washes with distilled water, the plates were air-dried and stained for 30 min with 0.4% (wt/vol) SRB in 1% acetic acid. After four washes with 1% acetic acid to remove the unbound dye, the plates were air-dried and cell-bound SRB was dissolved with 200 μl/well of 10 mM unbuffered Tris Base solution. The optical density (O.D.) of the samples was determined at 540 nm using a spectrophotometric plate reader. The percentage survival of the cultures treated with AT-101 was calculated by normalization of their OD values to those of control cultures treated with DMSO (Masuelli et al., 2017b). The experiments were performed in triplicate and repeated three times.

### Trypan Blue Exclusion Assay

For trypan blue exclusion assay, cells were seeded at 5 × 10^4^/well in 24-well plates and incubated at 37°C to allow cells attachment. After 24 h, the medium was changed and the cells were treated with AT-101 (1.56–3.13–6.25–12.5–25 μM) or DMSO and incubated for 24, 48, and 72 h. After 24, 48, and 72 h, adherent as well as suspended cells of each well were harvested and stained with trypan blue (Sigma-Aldrich, Milan, Italy) and counted with an optic microscope (Strober, 2015). The experiments were repeated three times. The percentage of cells death was determined compared to the total number of cells (Benvenuto et al., 2015).

### Fluorescent Measurement of Reactive Oxygen Species (ROS)

Dichlorofluorescin diacetate (DCF-DA) was used to detect ROS production in MM cells. Briefly, 2.5 × 10^5^ cells were seeded into 6-well plates and incubated at 37°C to allow cells attachment before treatment. After two washings with PBS, cells were incubated with 10 μM 2′,7′-dichlorofluorescein diacetate (Sigma-Aldrich, Milan, Italy) in PBS at 37°C and 5% CO_2_ in the dark for 30 min (Masuelli et al., 2016). After two washings, cells were treated with AT-101 (dose range: 1.56–25 μM) or DMSO in serum-free medium and incubated at 37°C and 5% CO_2_ in the dark for different times (15 min–4 h). Then, adherent and suspended cells were harvested, centrifuged at 1250 rpm for 10 min, and seeded in 96-well plates (100 μl per well). Fluorescence intensity was measured after 15 and 30 min and after 1 and 4 h using a spectrophotometric plate reader at an excitation wavelength of 495 nm and an emission wavelength of 535 nm. Because the highest level of fluorescence was detected at 30 min and it decreased back to the level of the control after 1 h of AT-101 stimulation (data not shown), this experimental time was chosen for subsequent experiments.

### FACS Analysis

Asynchronized, log-phase growing cells (60% confluent, about 2.5 × 10^5^/well in 6-well plates) were treated with AT-101 (3.13–6.25–12.5–25 μM) or DMSO in complete culture medium. Z-VAD-FMK was used at a final concentration of 40 μM for 2 h before addition of AT-101 treatment. After 48 h adherent as well as suspended cells were harvested, centrifuged at 1500 rpm for 10 min and washed twice with cold phosphate buffered saline (PBS). Cell pellets were resuspended in 70% ethanol and incubated for 1 h at -20°C. Cells were then washed twice with cold PBS, centrifuged at 1500 rpm for 10 min, incubated for 1 h in the dark with propidium iodide (25 μg/ml final concentration in 0.1% citrate and 0.1% Triton X-100) and analyzed by flow cytometry using a FACSCalibur cytometer through CellQuest software (Masuelli et al., 2012).

### Preparation of Cell Lysates and Western Blotting

About 1 × 10^6^ cells were seeded in 100 mm tissue culture dishes 24 h prior to the addition of 12.5 μM AT-101 or vehicle control. After 48 h of incubation, the cells were harvested, washed twice with cold PBS and lysed in RIPA lysis buffer (Triton X-100 1%, SDS 0.1%, NaCl 200 mM, Tris HCl 50 mM pH 7.5, PMSF 1 mM, NaOV 1 mM). After 30 min at 4°C, the mixtures were centrifuged at 12,000 *g* for 15 min and the supernatants were analyzed by Western blotting. For immunoblotting analysis, 80 μg of cell lysates were resolved in 10% SDS-PAGE and then transferred to nitrocellulose membranes. After blocking, the membranes were incubated with specific primary antibodies at the concentration of 1–2 μg/ml overnight at 4°C. After washing, the filters were incubated with goat anti-mouse or -rabbit IgG, peroxidase-conjugated antibodies and developed by chemiluminescence as previously described (Masuelli et al., 2013). Densitometric analysis of autoradiographic bands was performed using the Image J software (National Institutes of Health, United States) after blot scanning.

### Transmission Electron Microscopy

Ultrastructural analyses were performed on cells treated with 12.5 μM AT-101 or with DMSO for 24 h. After treatment, the cells were fixed in 2.5% glutaraldehyde in PBS pH 7.4, and the samples were processed for transmission electron microscopy following routine procedures (Masuelli et al., 2014).

### *In vivo* Treatment of C57BL/6 Mice Intraperitoneally Administered With AT-101 and Transplanted With #40a Cells

Groups of 6-to-8-weeks-old C57BL/6 mice (8 mice for each group) were intraperitoneally (i.p.) inoculated with 0.2 ml of suspension containing 1 × 10^6^ #40a cells in phosphate-buffered saline (PBS) 1 week after pristane injection (500 μl). Mice were treated i.p. with AT-101 (0.1 mg dissolved in 400 μl of corn oil, 1 time per week), or corn oil (400 μl, 1 time per week).

The treatments were started simultaneously with the inoculation of MM cells. Isolation of the murine mesothelioma 40 cell line was previously described by Goodglick et al. (1997). The #40a cell line is derived from the 40 cell line after two passages in the peritoneal cavity following administration of pristane 1 week before cell transplant. The intraperitoneal injection of a mineral oil such as pristane was shown to induce inflammation in mice (Shacter et al., 1992).

Investigation has been conducted in accordance with the ethical standards and according to the Declaration of Helsinki. A veterinary surgeon was present during the experiments. The animal care both before and after the experiments was performed only by trained personnel. Mice were bred under pathogen-free conditions in the animal facilities of the University of Rome “Tor Vergata” and handled in compliance with European Union and institutional standards for animal research. The work was conducted with the formal approval of the local animal care committees (institutional and national), and animal experiments have been registered as legislation requires (Authorization from Ministry of Health no. 187/2016-PR).

### Analysis of Antitumor Activity *in vivo*

#40a cells growth in the peritoneum induces ascites. Accordingly, the abdominal circumference of mice was monitored before the inoculation of cells and every week until tumor-bearing mice were euthanized at the first signs of distress or when their abdominal circumference exceeded 12 cm.

### Statistical Analysis

Data distribution of cell survival, cell death, ROS production and FACS analyses were preliminarily verified by Kolmogorov–Smirnov test, and data sets were analyzed by one-way analysis of variance (ANOVA) followed by Newman–Keuls test.

Differences in the intensity of immunoreactive bands were evaluated by a two-tailed Student’s *t*-test. Values with *p* ≤ 0.05 were considered significant.

Survival curves and tumor volumes were estimated using the Kaplan–Meier method and compared with a log-rank test (Mantel–Cox). Differences in tumor volumes were regarded as significant when the *p*-value was ≤0.05 (Benvenuto et al., 2016a).

## Results

### Effect of AT-101 on MM Cells Survival

The survival of human (MM-B1, H-Meso-1, and MM-F1) and mouse (#40a) MM cells was evaluated by the SRB assay after exposure to increasing doses of AT-101 (1.56–3.13–6.25–12.5–25 μM) or vehicle control (DMSO) for 24, 48, and 72 h. Experiments were also performed in #40a cells in order to establish whether this mouse cell line was susceptible to the *in vitro* anti-tumoral activity of AT-101 and thus it could be used as transplantable tumor cell line to determine the *in vivo* anti-tumoral effect of this polyphenol in C57BL/6 mice.

**FIGURE 1:**
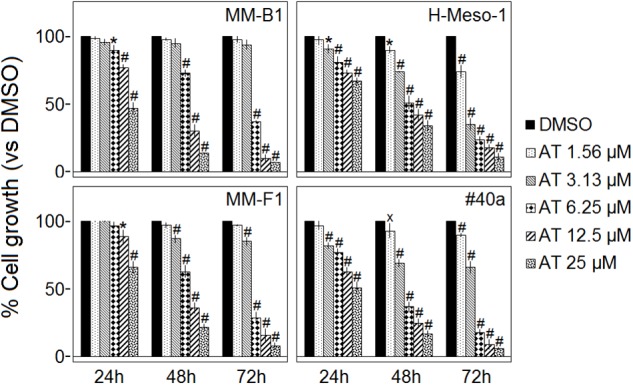
Effect of AT-101 (AT) on MM cell lines survival. The survival of human (MM-B1, H-Meso-1, and MM-F1) and mouse (#40a) MM cell lines were assessed by the SRB assay after 24, 48, and 72 h of treatment with DMSO or AT-101. The percentage of surviving cells treated with the compound was calculated by normalizing the OD value to that of the control cultures (DMSO). The results are expressed as the means ± SD of three independent experiments performed in triplicate (^x^*p* ≤ 0.05, ^∗^*p* ≤ 0.01, ^#^*p* ≤ 0.001 compared with the cultures treated with DMSO).

The effect of AT-101 on cell proliferation was dose- and time-dependent and gained statistical significance at doses from 6.25 to 25 μM in all MM cell lines, as compared to vehicle control, after 48 and 72 h of treatment. AT-101 significantly decreased the cells survival at all doses in H-Meso-1 and #40a cell lines after 48 and 72 h of treatment (Figure 1).

The concentrations of AT-101 required to reduce the cells survival by 50% (IC50) after 48 and 72 h were 9.30 and 5.59 μM for MM-B1, respectively; 9.24 and 2.66 μM for H-Meso-1, respectively; 9.32 and 5.10 μM for MM-F1, respectively; 5.44 and 3.87 μM for #40a, respectively (Table 1).

**Table 1:** AT-101 concentrations required for 50% inhibition of MM cell lines survival (IC50).

MM cell lines	AT-101 treatment (hours)	IC50 ± SD (μM)
MM-B1	48	9.30 ± 0.29
	72	5.59 ± 0.07
MM-F1	48	9.32 ± 0.70
	72	5.10 ± 0.45
H-Meso-1	48	9.24 ± 1.43
	72	2.66 ± 0.36
#40a	48	5.44 ± 0.25
	72	3.87 ± 0.24


### Effect of AT-101 on MM Cells Death

The cell death of human (MM-B1, H-Meso-1, and MM-F1) and mouse (#40a) MM cells was evaluated by the Trypan blue exclusion assay after the exposure to increasing doses of AT-101 (1.56–3.13–6.25–12.5–25 μM) or vehicle control (DMSO) for 24, 48, and 72 h. The dye exclusion assay was used to determine the number of viable MM cells upon AT-101 exposure.

**FIGURE 2:**
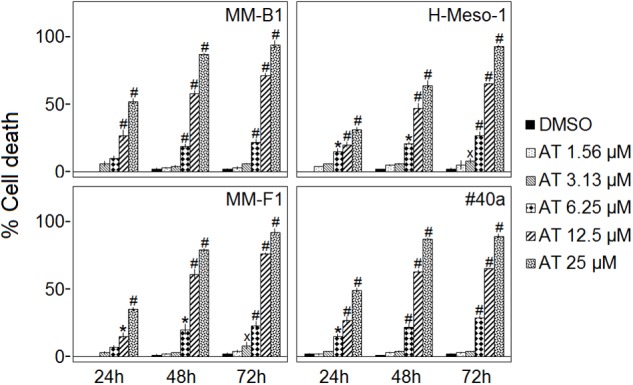
Effect of AT-101 (AT) on death of MM cell lines. Trypan blue exclusion assay was performed to determine the percentage of cell death of MM cells treated with AT-101 or DMSO after 24, 48, and 72 h of treatment. The results are expressed as the means ± SD of three independent experiments performed in triplicate (^x^*p* ≤ 0.05, ^∗^*p* ≤ 0.01, ^#^*p* ≤ 0.001 compared with the cultures treated with DMSO).

AT-101 significantly increased the percentage of cells death in a dose- and time-dependent manner in all MM cell lines as compared to vehicle control after 24, 48, and 72 h. The percentages of the cells death after AT-101 treatment were 94, 71, and 22 for MM-B1 (*p* < 0.001); 93, 65, and 27 for H-Meso-1 (*p* < 0.001); 92, 76, and 23 for MM-F1 (*p* < 0.001) and 89, 65, and 29 for #40a (*p* < 0.001) cells after 72 h of exposure at the highest doses (Figure 2).

### Effect of AT-101 on MM Cells ROS Production

One of the major detrimental effects of polyphenols on cancer cells is their ability to increase ROS production (Benvenuto et al., 2016b). To determine the effect of increasing doses of AT-101 (1.56–25 μM) on intracellular ROS production, the DCF-DA assay was performed in AT-101-treated MM cells. The effect of AT-101 was compared to that of DMSO and the results were expressed as the mean of the fluorescence intensity.

AT-101 did not influence the ROS production in MM-B1, H-Meso-1 and #40a cells compared to DMSO-treated cells. However, AT-101 increased significantly the ROS production only at the higher dose in MM-F1 cells as compared to the vehicle (Table 2).

**Table 2:** Effects of AT-101 (AT) on the intracellular ROS production in MM cell lines.

	MM-B1		MM-F1		H-Meso-1		#40a	
				
	Mean^a^	*p*	Mean	*p*	Mean	*p*	Mean	*p*
DMSO	199603		265236		193284		269566	
AT 1.56 μM	173255		222363		181133		242393	
AT 3.13 μM	177785		246219		201532		258737	
AT 6.25 μM	206563		280478		190579		242218	
AT 12.5 μM	221189		257207		214424		232550	
AT 25 μM	188801		308522	<0.05	197155		281290	


*^*a*^The results are reported as the mean of the fluorescence intensity values from three experiments performed in triplicate. AT-101 (AT) was used in the range 1.56–25 μM. Statistical significance of the effects obtained with AT-101 was calculated vs. those obtained in DMSO-treated cells with one-way ANOVA analysis of variance*.

### Effect of AT-101 on MM Cells Apoptosis and Cell Cycle Distribution

In order to evaluate the effect of AT-101 on apoptosis and cells cycle distribution, FACS analysis of DNA content was performed on MM cells treated with increasing doses of AT-101 (3.13–25 μM) for 48 h. DMSO was used as a vehicle.

AT-101 treatment induced an increase of the percentage of cells in the subG1 phase at 25 μM in H-Meso-1 cells, at 25–12.5 μM in MM-B1 cells, and at 25–12.5–6.25 μM in MM-F1 cells. The increase of the percentage of the cells in the subG1 phase was associated with a reduction of that in G0/G1, S and G2/M phases in all human MM cells at the highest dose, and at 12.5–6.25 μM for MM-F1 cells. The increase of the percentage of cells in the sub-G1 phase was associated with an increase of cells in G0/G1 phase and a decrease of cells in G2/M phase at 12.5 μM for MM-B1 cell line. We observed an increase of the number of cells in the subG1 phase only at the highest dose of AT-101 in #40a cells. This increase was associated with a dose-dependent increase of the percentage of cells in G2/M phase and a decrease of the number of cells in G0/G1 phase. A dose-dependent increase of the percentage of cells in G2/M phase and a decrease of cells in G0/G1 phase was observed for the other doses of AT-101 in #40a cells (Table 3).

**Table 3:** Effects of AT-101 (AT) alone or with the inhibitor of apoptosis Z-VAD-FMK on cell cycle distribution in MM cell lines after 48 h of treatment.

	μM	subG1^a^	*p^∗^*	G0/G1	*P*	*S*	*p*	G2/M	*p*
MM-B1	DMSO	0.69 ± 0.10		49.42 ± 2.09		18.54 ± 2.44		31.81 ± 4.34	
	AT 3.13	1.21 ± 0.13		57.48 ± 0.59	<0.001	17.50 ± 0.36		24.17 ± 0.11	<0.05
	AT 6.25	1.45 ± 0.12		59.63 ± 0.12	<0.001	16.93 ± 0.10		22.37 ± 0.16	<0.05
	AT 12.5	9.01 ± 0.06	<0.001	64.65 ± 0.45	<0.001	15.05 ± 0.45		11.68 ± 0.10	<0.001
	AT 25	64.40 ± 0.64	<0.001	29.59 ± 0.57	<0.001	3.81 ± 0.19	<0.001	2.61 ± 0.21	<0.001
	AT25 + Z-VAD	15.30 ± 6.16	<0.01	64.80 ± 3.95	<0.01	13.17 ± 1.58	<0.05	7.13 ± 0.64	<0.05
MM-F1	DMSO	1.79 ± 0.10		70.49 ± 1.59		6.39 ± 0.11		21.71 ± 1.78	
	AT 3.13	2.88 ± 0.11		67.16 ± 1.72		7.07 ± 0.94		23.28 ± 2.74	
	AT 6.25	10.37 ± 0.42	<0.001	61.31 ± 2.44	<0.01	6.33 ± 0.27		22.44 ± 3.12	
	AT 12.5	24.91 ± 1.06	<0.001	48.81 ± 1.28	<0.001	7.74 ± 1.01		18.99 ± 3.32	
	AT 25	73.38 ± 0.08	<0.001	17.64 ± 0.71	<0.001	3.01 ± 0.13	<0.01	6.13 ± 0.74	<0.01
	AT25 + Z-VAD	29.70 ± 0.74	<0.001	51.76 ± 0.21	<0.001	6.45 ± 0.18	<0.01	12.46 ± 1.12	<0.05
H-Meso-1	DMSO	2.14 ± 0.41		52.63 ± 1.06		13.96 ± 1.41		31.69 ± 2.87	
	AT 3.13	2.41 ± 0.40		54.61 ± 0.41		12.98 ± 0.10		30.35 ± 0.75	
	AT 6.25	4.02 ± 1.03		51.33 ± 0.40		12.07 ± 0.54		32.93 ± 0.92	
	AT 12.5	6.70 ± 1.05		50.80 ± 1.62		13.06 ± 0.26		29.81 ± 0.78	
	AT 25	38.82 ± 2.94	<0.001	36.82 ± 1.41	<0.001	9.76 ± 0.47	<0.05	14.97 ± 1.08	<0.001
	AT25 + Z-VAD	23.93 ± 3.42	<0.05	45.49 ± 1.15	<0.05	11.63 ± 0.20	<0.05	19.34 ± 2.06	
#40a	DMSO	1.62 ± 0.10		55.90 ± 0.10		12.43 ± 0.40		30.50 ± 0.59	
	AT 3.13	1.39 ± 0.62		51.17 ± 0.79	<0.001	12.34 ± 0.38		35.49 ± 0.21	<0.05
	AT 6.25	1.55 ± 0.62		34.38 ± 0.78	<0.001	15.29 ± 0.33		49.26 ± 0.18	<0.001
	AT 12.5	2.18 ± 0.41		2.18 ± 0.11	<0.001	7.32 ± 2.59		88.59 ± 2.27	<0.001
	AT 25	5.48 ± 0.10	<0.001	3.96 ± 0.64	<0.001	12.16 ± 2.51		78.87 ± 3.10	<0.001
	AT25 + Z-VAD	5.38 ± 0.47		4.72 ± 0.89		13.56 ± 1.24		76.82 ± 2.49	


^a*^Percentage of cells in subG1, G0/G1, S, and G2/M phases was calculated with CellQuest Pro 5.2 software. The results reported are mean values from three independent experiments. ^∗^Significance of the effect of AT-101 (AT) was calculated vs. that of DMSO-treated cells with one-way ANOVA analysis of variance. Significance of the effect of AT-101 25 μM + Z-VAD was calculated vs. that of AT-101 25 μM employing the Student’s t-test.

The subG1 phase is characterized by the hypodiploid DNA content, typical of apoptosis. To confirm the effect of AT-101 in inducing MM cells apoptosis, MM cells were simultaneously exposed to AT-101 and to the Z-VAD-FMK, a universal inhibitor of caspases. Z-VAD-FMK was able to significantly reduce AT-101-mediated apoptosis in all human MM cell lines, suggesting the apoptotic effect of the AT-101 treatment in MM cells (Table 3).

To corroborate that AT-101 treatment induced apoptosis, the expression of p53, as well as Bax/Bcl-2 expression ratio was analyzed by Western blotting. Human and mouse MM cell lines were treated with 12.5 μM AT-101 for 48 h. Overall, results obtained were found to be dependent on the cell line investigated.

**FIGURE 3:**
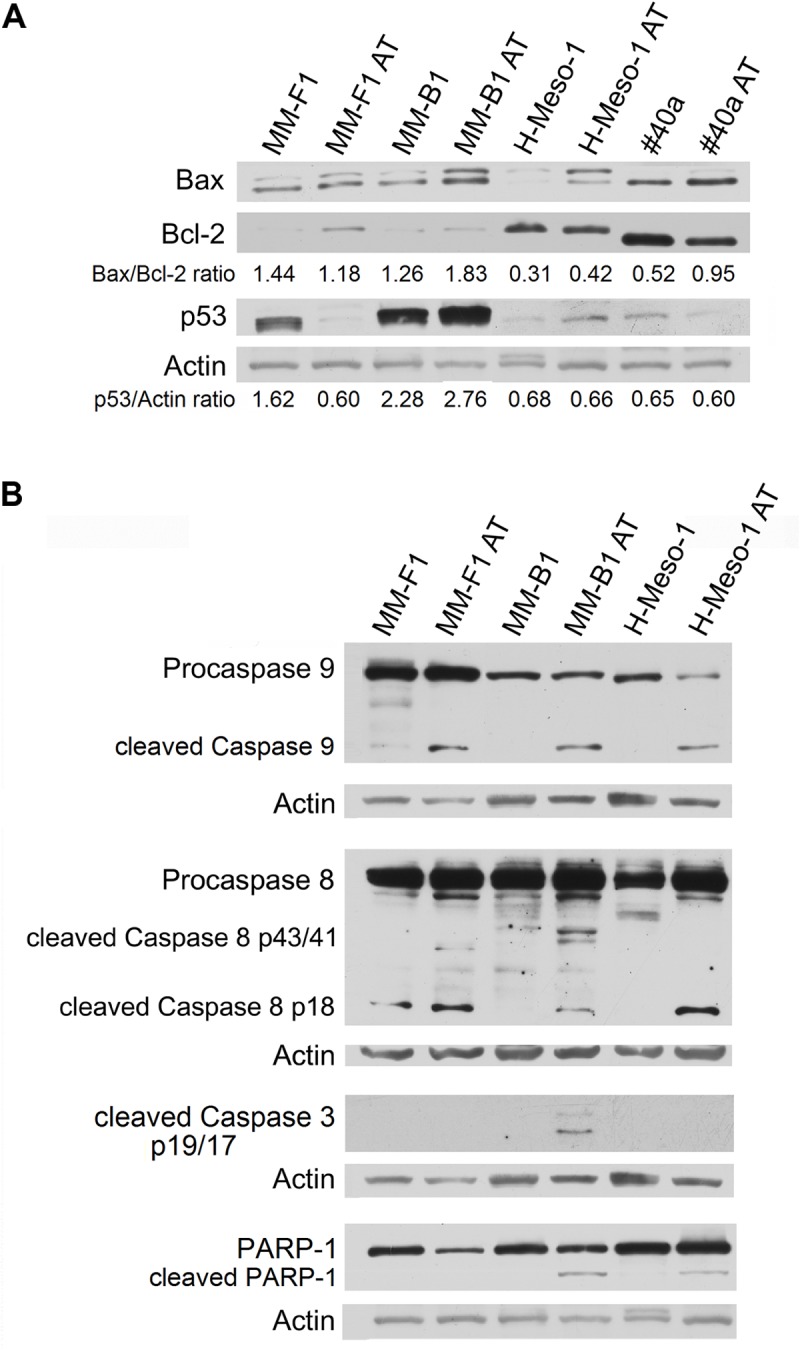
Effect of AT-101 on the expression of molecules involved in apoptosis in MM cells. **(A)** The expression of Bax, Bcl-2 and p53 was assessed by Western blotting analysis in MM cells treated for 48 h with AT-101 at 12.5 μM or with DMSO as vehicle control. Actin was used as an internal control. The intensity of the bands obtained from two independent experiments was quantified using ImageJ software after blot scanning, and the densitometric ratios are reported. **(B)** MM cells were treated with AT-101 and the expression of procaspases (9-8), cleaved caspases (9-8-3) and the cleavage of PARP-1 were analyzed by western blotting. Actin was used as an internal control. The intensities of the bands obtained from two independent experiments were quantified using ImageJ software after blot scanning.

The AT-101 treatment significantly increased the Bax/Bcl-2 ratio in MM-B1, H-Meso-1 and #40a cells compared to DMSO treatment (MM-B1, *p* = 0.014; H-Meso-1, *p* = 0.004; #40a, *p* = 0.0002) and down-regulated the expression of Bcl-2 in H-Meso-1 (*p* = 0.005) and #40a (*p* = 0.029) cells. Conversely, AT-101 increased the expression of Bcl-2 (*p* = 0.009), but not that of Bax, causing a decrease of the Bax/Bcl-2 ratio in MM-F1 cells (*p* = 0.030) (Figure 3A).

In addition, AT-101 increased p53 expression in MM-B1 (*p* = 0.0006), while decreased it in MM-F1 (*p* = 0.012) cells. p53 expression did not change after AT-101 treatment in H-Meso-1 and #40a cells (Figure 3A).

We investigated the effect of AT-101 on the activation of the intrinsic apoptotic pathway. The activation of this pathway is sustained by the activation of the procaspase 9 into caspase 9. All AT-101-treated human MM cells showed the activation of procaspase 9 as detected by the presence of caspase 9 cleavage fragment of about 35 kDa (Figure 3B).

In addition, AT-101 was able to induce the activation of the extrinsic pathway of apoptosis, as demonstrated by the proteolytic cleavage of caspase 8 into the active fragments p43/41 and/or p18 in all human MM cell lines (Figure 3B).

Activated caspases 8 and 9 are able to cleave and activate caspase 3, that induces the proteolytic inactivation of poly (ADP-ribose) polymerase-1 (PARP-1), which impairs DNA repair and genomic integrity (Kaufmann et al., 1993). Our results showed that AT-101 induced the proteolytic cleavage of caspase 3 into the activated fragments p19 and p17 in MM-B1 cells. In addition, AT-101 mediated the proteolytic cleavage of PARP-1 in MM-B1 and H-Meso-1 cells. Although no cleavage of PARP-1 was detected, we observed a decreased expression of PARP-1 (*p* = 0.0002) in MM-F1-treated cells (Figure 3B).

### Effect of AT-101 on MM Cell Lines Autophagy

**FIGURE 4:**
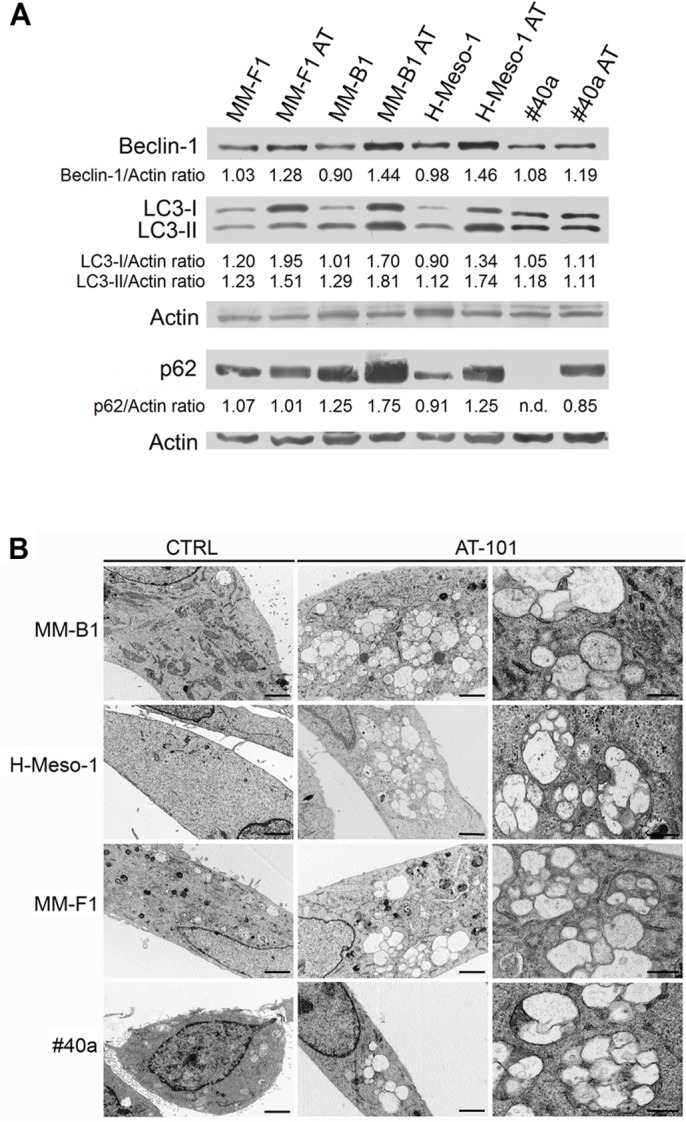
Effect of AT-101 on autophagy in MM cells. **(A)** The expression of Beclin-1, p62/SQSTM (p62), LC3-I, and LC3-II was assessed by Western blotting in MM cell lines treated with AT-101 at 12.5 μM or DMSO vehicle for 48 h. Actin was used as an internal control. The intensity of the bands obtained was quantified using the ImageJ software after blot scanning of two independent experiments. The densitometric ratios between Beclin-1 and actin, p62 and actin, LC3-I and actin, LC3-II and actin are reported. n.d., not detectable. **(B)** Ultrastructural analysis of autophagy in MM cells by transmission electron microscopy. Cells were treated with 12.5 μM AT-101 or DMSO for 24 h, fixed in glutaraldehyde, postfixed in osmium tetroxide and embedded in epon 812 resin. The experiment was repeated twice. Cytoplasmic autophagic vacuoles surrounded by a double membrane, containing cytoplasm, vesicles and mitochondria are visible in AT-101 treated cells. Bars correspond to 1 μm (first and second column) or to 200 nm (third column).

AT-101 is a BH3 mimetic compound able to interrupt the interaction between Beclin-1 and Bcl-2. In this way, AT-101 activates Beclin-1-dependent autophagy (Marquez and Liang, 2012). Our results showed that AT-101 significantly increased Beclin-1 expression in all cell lines (MM-F1, *p* = 0.003; MM-B1, *p* = 0.0007; H-Meso-1, *p* = 0.0008; #40a, *p* = 0.048) (Figure 4A).

The activation of the autophagic process can be revealed by the conversion of the cytosolic form LC3-I into the membrane-bound form LC3-II. The amount of LC3-II correlates with the number of autophagosomes which are the markers of autophagy (Mizushima and Yoshimori, 2007). Our results showed that LC3-I and LC3-II were constitutively expressed in control MM cells. The treatment with AT-101 induced a significant increase of LC3-I (MM-F1, *p* = 0.0008; MM-B1, *p* = 0.0006; H-Meso-1, *p* < 0.0001) and also of LC3-II (MM-F1, *p* = 0.0003; MM-B1, *p* = 0.003; H-Meso-1, *p* = 0.0006) in all human MM cell lines, while the expression of these proteins remained unchanged in #40a cells (Figure 4A).

In addition, AT-101 induced the increase of p62 in MM-B1 (*p* = 0.001), H-Meso-1 (*p* = 0.003) and #40a (*p* < 0.0001) cells, while the expression of p62 did not change in MM-F1 cells (Figure 4A).

These results suggest that AT-101 induced autophagy, but the process was then blocked in MM-B1, H-Meso-1, and #40a cells as indicated by the increase of p62.

Induction of autophagy was confirmed by transmission electron microscopy (Barth et al., 2010). Ultrastructural analysis was performed on MM-B1, MM-F1, H-Meso-1, and #40a cells treated with 12.5 μM of AT-101 or with DMSO for 24 h. After treatment, cells were fixed in 2.5% glutaraldehyde in PBS pH 7.4 at 4°C and processed for electron microscopy observation. As shown in Figure 4B, AT-101 was able to induce autophagy in all AT-101-treated MM cell lines compared to control cells. In detail, cells treated with AT-101 showed the presence of cytoplasmic autophagic vacuoles surrounded by a double membrane, containing cytoplasm, vesicles, and mitochondria (Figure 4B).

### Effect of AT-101 on MM Cell Lines Expression and Activation of ErbB Receptors and Pro-survival Signaling Pathway Members

One of the signal transduction pathways activated in mesothelial cells by asbestos is that of ErbB receptors (Heintz et al., 2010). Thus, we evaluated the expression of EGFR and ErbB2, the expression and phosphorylation of MAPKs, including ERK1/2, the p38 kinase and the c-Jun N-terminal kinases (JNKs p54 and p46) by Western blotting after treatment of MM cells with AT-101 at 12.5 μM for 48 h. Also for this analysis, results were found to be dependent on the cell line analyzed.

**FIGURE 5:**
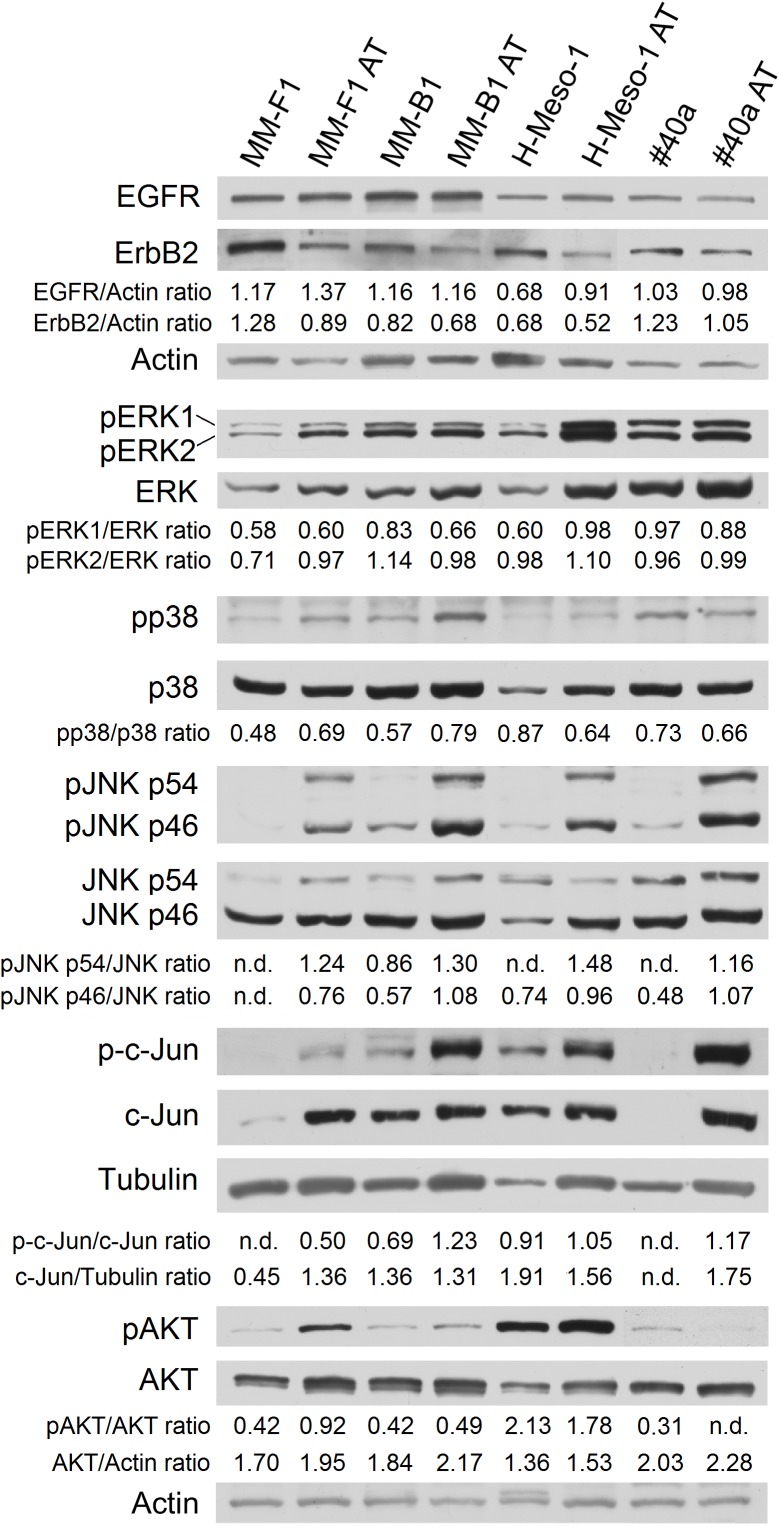
Effect of AT-101 on the expression and activation of signaling pathway molecules. Western blotting analysis was performed on MM cells treated with AT-101 (12.5 μM) or DMSO vehicle for 48 h. The levels of pERK1 and pERK2 proteins, as well as p-p38 protein, as well as pJNK, as well as p-c-Jun, as well as pAKT, were compared with that of total ERK, p38, JNK, c-Jun, and AKT proteins, respectively. The intensity of the bands was quantified using the ImageJ software after blot scanning of two independent experiments. The ratios are reported. Actin and tubulin were used as an internal control. n.d, not detectable.

As shown in Figure 5, AT-101 reduced the expression of ErbB2 in all MM cell lines (MM-F1, *p* = 0.004; MM-B1, *p* = 0.002; H-Meso-1, *p* = 0.003; #40a, *p* = 0.0003) as compared to DMSO-treated cells. On the other hand, AT-101 increased EGFR expression in MM-F1 (*p* = 0.007) and H-Meso-1 (*p* = 0.0004) cells, while the expression remained unchanged in the other cell lines (Figure 5).

In addition, AT-101 decreased the level of phosphorylation of ERK1 in MM-B1 cells (*p* = 0.0002), while increased that of ERK1 in H-Meso-1 cells (*p* = 0.008) and that of ERK2 in MM-F1 cells (*p* = 0.03) (Figure 5).

p38 phosphorylation was increased after AT-101 treatment in MM-F1 (*p* = 0.014) and MM-B1 (*p* = 0.012) cells, while the effect was opposite in H-Meso-1 (*p* = 0.014) and #40a (*p* = 0.021) cells (Figure 5).

All AT-101-treated cells showed the increased phosphorylation of p54 (MM-F1, *p* < 0.0001; MM-B1, *p* = 0.002; H-Meso-1, *p* < 0.0001; #40a, *p* = 0.0002) and p46 (MM-F1, *p* = 0.001; MM-B1, *p* = 0.0008; H-Meso-1, *p* = 0.02; #40a, *p* = 0.006) JNK compared to DMSO-treated cells. Furthermore, AT-101 significantly increased the expression of c-Jun in MM-F1 (*p* = 0.002) and #40a (*p* < 0.0001) cells, and the c-Jun phosphorylation in all MM cells (MM-F1, *p* = 0.002; MM-B1, *p* = 0.007; H-Meso-1, *p* = 0.016; #40a, *p* = 0.001) (Figure 5).

Finally, we evaluated whether AT-101 treatment inhibited the expression and phosphorylation of the pro-survival kinase AKT, which promotes tumor growth. Our results showed that AT-101 decreased AKT phosphorylation in H-Meso-1 (*p* = 0.03) and #40a (*p* < 0.0001) cells, while increased it in MM-F1 (*p* < 0.0001) and MM-B1 (*p* = 0.002) cells (Figure 5).

### Effect of AT-101 on Tumor Growth in C57BL/6 Mice Intraperitoneally Transplanted With #40a Cells

We analyzed the *in vivo* anti-tumor effects of AT-101 employing C57BL/6 mice (8 mice per group) intraperitoneally inoculated with 1 × 10^6^ #40a cells. These mice were simultaneously, intraperitoneally administered with 0.1 mg of AT-101 dissolved in corn oil or with the vehicle alone (CTR). The treatment was performed once a week. The measurement of the abdominal circumference of the mice was assessed prior to cells inoculation and then every week to monitor the growth of #40a cells which induced ascites.

**FIGURE 6:**
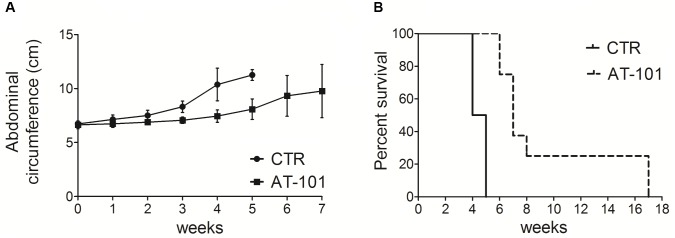
AT-101 reduced tumor growth and increased the survival in C57BL/6 mice intraperitoneally transplanted with MM #40a cells. **(A)** Differences in the mean abdominal circumferences between C57BL/6 mice treated with AT-101 or with corn oil (CTR). **(B)** Differences in the mean survival time of C57BL/6 mice treated with AT-101 or with corn oil (CTR). Eight mice each group were inoculated.

Mice treated with AT-101 showed a significant decrease in the abdominal circumference compared to control mice (mean value 6.9 cm compared with 7.5 cm, *p* = 0.0053) after 2 weeks of treatment (Figure 6A). All control mice were euthanized at 4 (four mice) and 5 (four mice) weeks of treatment, for the excessive tumor size. Conversely, all AT-101-treated mice remained alive at this time (mean abdominal circumference 8.1 cm). AT-101-treated mice were euthanized at 6 weeks (two mice), at 7 weeks (three mice) and at 8 weeks (one mouse) of treatment. Two AT-101-treated mice remained alive until 17 weeks, when they were euthanized for the excessive tumor size. The increase in the median survival of AT-101-treated mice was significant compared to vehicle-treated mice (7 vs. 4.5 weeks; *p* = 0.0001) (Figure 6B).

Overall, our results indicated specific interference with intraperitoneally transplanted MM #40a cells growth by AT-101. Indeed, when comparing the survival of C57BL/6 mice upon treatment, it was observed that the risk of developing tumors in the corn oil-treated mice was 25.19 relative to the AT-101-treated mice (Table 4).

**Table 4:** Analysis of the survival of C57BL/6 mice after treatment with AT-101 by the log-rank test (Mantel–Cox).

Variable	Contrast	Hazard ratio	95% Hazard ratio confidence limits		*p*-value	Median survival (weeks)
					
			Lower	Upper		
Treatment	CTR vs. AT-101	25.19	4.936	128.5	0.0001	4.5 vs. 7


*CTR, control*.

## Discussion

The survival of MM patients remains poor although surgery, chemotherapy and radiation have increased the quality of patient’s life (Favoni and Florio, 2011). One of the major drawback of the anti-cancer agents is that they show a poor bioavailability at the tumor sites by reducing the treatment efficacy (Al-Abd et al., 2015). The intra-tumoral administration of anti-cancer agents in the serous cavity could enhance their bioavailability at the tumor site, thus improving their efficacy (Antman, 1993). We recently demonstrated that curcumin and apigenin inhibited the growth of transplanted MM cells in the peritoneal cavity of mice when the compounds were administered intraperitoneally (Masuelli et al., 2017a,b). (-)-Gossypol (AT-101) was shown to have anti-tumoral activity by targeting multiple signaling transduction pathways in several tumors, including leukemia, lymphoma, colon carcinoma, breast cancer, myoma, prostate cancer and others (Keshmiri-Neghab and Goliaei, 2014). Several clinical trials employing AT-101 alone or in combination with different drugs have been developed and some trials are still ongoing (Opydo-Chanek et al., 2017; ClinicalTrials.gov, 2018).

Accordingly, we evaluated the *in vitro* and *in vivo* effects of AT-101 administration in MM. To our knowledge, this is the first study that investigated the *in vitro* and *in vivo* anti-tumoral effects of AT-101 in MM. Our data demonstrated that the treatment of human MM cell lines and murine #40a cells with AT-101 was able to inhibit the cell survival *in vitro* in a dose- and time-dependent manner.

AT-101 is a BH3 mimetic compound (Opydo-Chanek et al., 2017). The Bcl-2 family proteins (Bcl-2, Bcl-xL, Bcl-W, Mcl-1, A1/BFL-1) interact with BH3 proteins, such as Bax or Beclin-1, and regulate various intracellular pathways, including apoptosis and autophagy (Maiuri et al., 2007; Sinha and Levine, 2008; Vela et al., 2013; Benvenuto et al., 2017). It has been demonstrated that gossypol binds to the BH3 binding groove of anti-apoptotic Bcl-2 proteins, thus inhibiting the anti-apoptotic function of Bcl-2, Bcl-xl, and Mcl-1, and inducing apoptosis of cancer cells (Kang and Reynolds, 2009). In addition, AT-101 was shown to prevent the interaction between Bcl-2 and Beclin-1 at the endoplasmic reticulum, to decrease the levels of Bcl-2 and to increase Beclin-1 expression by inducing Beclin-1 Atg5-dependent autophagic pathway in cancer cells (Lian et al., 2011).

Here we demonstrated that AT-101 treatment was able to induce apoptosis and modulate cells cycle distribution in MM cells. AT-101 induced an increase in the percentage of cells in subG1 phase, representing the apoptotic cell population in MM cell lines after 48 h of treatment. The caspase inhibitor, Z-VAD-FMK was able to significantly reduce the number of cells in the subG1 phase, thus corroborating the induction of cell death by apoptosis following treatment with AT-101. Furthermore, we demonstrated that AT-101 was also able to activate Beclin-1-dependent autophagy. Previous studies reported the *in vitro* induction of autophagy following gossypol treatment in breast cancer (Gao et al., 2010), malignant glioma (Voss et al., 2010), melanoma (Jang and Lee, 2014), gastric cancer (Wei et al., 2016), head and neck cancer (Benvenuto et al., 2017), colon cancer (Lu et al., 2017). In our study, AT-101 stimulated autophagy, but the process was then blocked, as indicated by the increase of the levels of p62/SQSMT1 and was coincident with the activation of apoptosis in MM-B1, H-Meso-1 and #40a cells. Autophagy was activated only in MM-F1 cells, in which the levels of p62 remained unchanged after AT-101 treatment. Indeed, it has been reported that the levels of p62 are inversely correlated to autophagic activity (Lippai and Lőw, 2014) and that p62 is involved in the regulation of apoptosis by activating caspase 8 (Jin et al., 2009). Here, we provide evidence that AT-101 activates the extrinsic pathway of apoptosis, as indicated by the cleavage of caspase 8. This apoptotic pathway was activated also in MM-F1-treated cells, in which AT-101 triggered autophagy. We observed that AT-101 increased ROS production only in this cell line. It has been reported that one of the major detrimental effects of polyphenols on cancer cells is their ability to increase ROS production (Benvenuto et al., 2016b) and that increased levels of ROS could induce apoptosis and autophagy by the damage of DNA, proteins, and lipids (Zhang et al., 2015). We demonstrated that AT-101 was also able to induce the intrinsic pathway of apoptosis, by increasing the Bax/Bcl-2 expression ratio, by activating caspase 9 and by inducing the proteolytic cleavage of PARP-1. We observed only a significant down-regulation of the expression of total PARP-1 in MM-F1 cells. It has been reported that the decreased expression of PARP-1 could be the result of the degradation of PARP-1, without cleavage into the apoptotic fragments, mediated by caspase-independent ubiquitylation (Kim et al., 2015). Accordingly, the observed decrease of the expression of PARP-1 might confirm apoptosis activation in MM-F1 cells. Several studies have previously demonstrated that gossypol is able to activate both the intrinsic and extrinsic pathways of apoptosis in acute myeloid leukemia stem-like cells (Zhang et al., 2017), in mouse pituitary corticotroph tumor cells (Yurekli et al., 2018), in multiple myeloma cells (Lin et al., 2013), in human colorectal carcinoma cells (Ko et al., 2007) and in human alveolar lung cancer cells (Chang et al., 2004). In addition, we found that AT-101 treatment increased p53 expression only in MM-B1 cells, decreased it in MM-F1 cells, while p53 expression did not change in H-Meso-1 and #40a cells. Thus, the effect of the compound might be histotype-specific and AT-101 promoted apoptosis through p53-dependent or -independent pathway. Several studies reported that gossypol triggered apoptosis through the p53 pathway in several cancer cells (Volate et al., 2010; Hsiao et al., 2012; Karaca et al., 2013; Wei et al., 2016). On the other hand, it has been reported that gossypol-induced apoptosis was not be dependent on the response of p53 in colon cancer cells (Wang et al., 2000; Zhang et al., 2003) and in alveolar lung cancer cells (Chang et al., 2004). Accordingly, the induction of apoptosis by AT-101 was not associated with the regulation of p53 in H-Meso-1 and #40a cells, with an epithelial histotype. In addition, the decrease of the p53 expression associated with the activation of apoptosis in MM-F1 cells could be due to transcriptionally independent activities of p53 or to a ubiquitination or degradation of p53 after the activation of the intrinsic pathway of apoptosis (Comel et al., 2014).

Different signaling transduction pathways play a role in cell growth, autophagy, and apoptosis and the ErbB receptors pathway is activated in mesothelial cells by asbestos (Heintz et al., 2010). In our study, we demonstrated that AT-101 is able to interfere with the ErbB receptors pathway. We found that AT-101 reduced the expression of ErbB2 in all MM cell lines, while increased that of EGFR in MM-F1 and H-Meso-1 cells. In addition, AT-101 decreased the level of phosphorylation of ERK1 in MM-B1 cells while increased that of ERK1 in H-Meso-1 cells and that of ERK2 in MM-F1 cells. These opposite effects might be due to the expression of alternative pathways activation in the different histotypes of the MM cell lines thus reflecting the natural tumor heterogeneity occurring in human MM tumors. On the other hand, these cell lines were sensitive to the pro-apoptotic effect of AT-101 as well, thus suggesting that EGFR and MAPK activation could trigger cell death. Indeed, it has been shown that prolonged EGFR signaling, which might result from EGFR overexpression or defective EGFR signaling attenuation, could lead to the induction of apoptosis (Rush et al., 2012; Alanazi et al., 2015; Treda et al., 2016). It was also reported that activation of MAPK/ERK could protect or contribute to the cell death depending on the specific context (Zhuang and Schnellmann, 2006). Moreover, different polyphenols, including resveratrol, quercetin, apigenin, and taxol, induced cell death through the activation of ERK1/2 (Cagnol and Chambard, 2010).

Activation of JNK1/2 and p38-MAPK stress pathways is involved in the regulation of cell proliferation, differentiation, and apoptosis (Wada and Penninger, 2004; Lei et al., 2014). p38 phosphorylation was increased after AT-101 treatment in MM-F1 and MM-B1 cells, while the effect was opposite in cells with epithelial histotype H-Meso-1 and #40a. It has been reported that some chemotherapeutic agents, including polyphenols, require p38 activation for the induction of apoptosis or autophagy (Sui et al., 2014). On the other hand, it has been shown that p38 signaling enhances cells survival and growth (Wada and Penninger, 2004). A study reported that activation of the JNK pathway by gossypol was required for the induction of apoptosis in head and neck squamous cell carcinoma and in human leukemic cells (Zerp et al., 2009). In addition, we previously demonstrated that gossypol-induced apoptosis was mediated by the activation of JNK and p38 in head and neck cancer cells (Benvenuto et al., 2017). Here we showed an increased expression of p54 and p46 JNK phosphorylation in all AT-101-treated cells. JNK mediates the phosphorylation of c-Jun (Johnson and Nakamura, 2007). AT-101 significantly increased the expression of c-Jun in MM-F1 and #40a cells, and the c-Jun phosphorylation in all MM cells.

Finally, we showed that AT-101 affected the expression and phosphorylation of the pro-survival kinase AKT, which promotes tumor growth, depending on the cell type. AT-101 decreased AKT phosphorylation in H-Meso-1 and #40a cells, while increased it in MM-F1 and MM-B1 cells. However, despite the activation of AKT in these cell lines, we also observed activation of apoptosis. It has been reported that AKT could be activated in response to an apoptotic signal (Tang et al., 2001).

Several *in vivo* studies investigated the anti-tumoral activity of gossypol in different types of cancer. Loberg et al. (2007) demonstrated that oral administration of AT-101 (15 mg/kg) in combination with surgical castration significantly reduced the growth of prostate cancer induced by a subcutaneous injection of VCaP cells in SCID mice. Anti-tumoral activity of orally (-)-gossypol (20–40 mg/kg) in a mouse model of medulloblastoma has been reported (Wang et al., 2015). Xu et al. (2005) observed that oral administration of gossypol (10 mg/kg) was safe in animals and enhanced the anti-tumor activity of X-ray irradiation in a xenograft model of prostate cancer in nude mice. It has been reported that the intraperitoneal injection of (-)-gossypol (5-10 mg/kg, daily, for 7 days) or the intralesional injection of (-)-gossypol (15 mg/kg, daily) significantly suppressed tumor growth in the same murine model (Zhang et al., 2010; Pang et al., 2011). In addition, it has been observed that oral administration of AT-101 (35 mg/kg) and gefitinib (50 mg/kg) increased the tumor regression compared to single treatments in a mouse model of non-small cell lung cancer (Zhao et al., 2016). Here, we analyzed for the first time the effect of the intratumoral administration of AT-101 in an *in vivo* model of MM tumor growth. We demonstrated that AT-101 treatment was able to significantly reduced peritoneal #40a MM cells growth in C57BL/6 mice. The risk of developing tumors in the corn oil-treated mice was 25.19 in comparison to those treated weekly with 5 mg/kg AT-101. AT-101 prolonged the median survival of mice. The increase in the median survival of mice administered with AT-101 was superior to that of mice receiving corn oil (7 vs. 4.5 weeks; *p* = 0.0001). Overall, our results indicated a specific interference by AT-101 with the growth of intraperitoneally transplanted MM #40a cells.

Overall, our results provided evidence that the treatment with AT-101 is capable of inhibiting cell proliferation by targeting several signaling pathways, inducing apoptosis of human and murine MM cell lines and interfering with the *in vivo* tumor growth of MM #40a cells transplanted into the peritoneum of C57BL/6 mice.

Malignant mesothelioma is an aggressive tumor, thus the ability of AT-101 to interfere with the *in vivo* growth of MM cells could offer an additional tool for the treatment of this tumor. Therefore, the development of targeted therapies with BH3 mimetics, such as AT-101, could be a promising strategy to improve the effectiveness of standard therapies for patients with MM.

## Author Contributions

MB performed cell proliferation, western blotting and FACS analyses, statistical analysis, and wrote the manuscript. RM performed cell death experiments, ROS production, western blotting analysis, and *in vivo* experiments. JS performed *in vivo* experiments. PR, CC, MG, AV, and AM critically revised the manuscript. LM performed ultrastructural analysis and analyzed the results. RB supervised the project, analyzed the results, and wrote the manuscript.

## Conflict of Interest Statement

The authors declare that the research was conducted in the absence of any commercial or financial relationships that could be construed as a potential conflict of interest.
